# Persons with first episode psychosis have distinct profiles of social cognition and metacognition

**DOI:** 10.1038/s41537-021-00187-8

**Published:** 2021-12-09

**Authors:** M. Ferrer-Quintero, D. Fernández, R. López-Carrilero, I. Birulés, A. Barajas, E. Lorente-Rovira, L. Díaz-Cutraro, M. Verdaguer, H. García-Mieres, J. Sevilla-Llewellyn-Jones, A. Gutiérrez-Zotes, E. Grasa, E. Pousa, E. Huerta-Ramos, T. Pélaez, M. L. Barrigón, F. González-Higueras, I. Ruiz-Delgado, J. Cid, S. Moritz, Spanish Metacognition Group, S. Ochoa

**Affiliations:** 1 Parc Sanitari Sant Joan de Déu, Sant Boi de Llobregat (Barcelona), Barcelona, Spain; 2 Departament de Psicologia Social i Psicologia Quantitativa, Universitat de Barcelona, Barcelona, Spain; 3 Investigación Biomédica en Red de Salud Mental (CIBERSAM), Barcelona, Spain; 4 Serra Húnter fellow. Department of Statistics and Operations Research (DEIO), Universitat Politècnica de Catalunya · BarcelonaTech (UPC), Barcelona, Spain; 5 Institute of Mathematics of UPC - BarcelonaTech (IMTech), Barcelona, Spain; 6 Fundació Sant Joan de Déu, Esplugues de Llobregat (Barcelona), Barcelona, Spain; 7 Departament de Psicologia Clínica i de la Salut, Facultat de Psicologia, Universitat Autònoma de Barcelona, Bellaterra, Cerdanyola del Vallès, Barcelona, Spain; 8 Department of Research, Centre d’Higiene Mental Les Corts, Barcelona, Spain; 9 Psychiatry Service, Hospital Clínico Universitario de Valencia, Barcelona, Spain; 10 Institute of Psychiatry and Mental Health, Health Research Institute (IdISSC), Clinico San Carlos Hospital, Madrid, Spain; 11 Hospital Universitari Institut Pere Mata, Institut d’Investigació Sanitària Pere Virgili (IISPV), Universitat Rovira i Virgili, Reus, Spain; 12 Department of Psychiatry, Hospital de la Santa Creu i Sant Pau, Institut d’Investigació Biomèdica-Sant Pau (IIB-Sant Pau), Universitat Autònoma de Barcelona, Barcelona, Spain; 13 Salut Mental Parc Taulí. Sabadell (Barcelona), Hospital Universitari – UAB Universitat Autònoma de Barcelona, Barcelona, Spain; 14 Neuropsiquiatria i Addicions, Hospital del Mar, IMIM (Hospital del Mar Medical Research Institute), Barcelona, Spain; 15 Department of Psychiatry, IIS-Fundación Jiménez Díaz Hospital (Madrid), Madrid, Spain; 16 Psychiatry Service, Area de Gestión Sanitaria Sur Granada, Motril (Granada), Spain; 17 Comunidad Terapéutica Jaén Servicio Andaluz de Salud, Jaén, Spain; 18 Unidad de Salud Mental Comunitaria Malaga Norte, Málaga, Spain; 19 Mental Health & Addiction Research Group, IdiBGi, Institut d’Assistencia Sanitària, Girona, Spain; 20 Department of Psychiatry and Psychotherapy, University Medical Center Hamburg, Hamburg, Germany

**Keywords:** Health sciences, Psychosis, Schizophrenia

## Abstract

Subjects with first-episode psychosis experience substantial deficits in social cognition and metacognition. Although previous studies have investigated the role of profiles of individuals in social cognition and metacognition in chronic schizophrenia, profiling subjects with first-episode psychosis in both domains remains to be investigated. We used latent profile analysis to derive profiles of the abilities in 174 persons with first-episode psychosis using the Beck’s Cognitive Insight Scale, the Faces Test, the Hinting Task, the Internal, Personal and Situational Attributions Questionnaire, and the Beads Task. Participants received a clinical assessment and a neuropsychological assessment. The best-fitting model was selected according to the Bayesian information criterion (BIC). We assessed the importance of the variables via a classification tree (CART). We derived three clusters with distinct profiles. The first profile (33.3%) comprised individuals with low social cognition. The second profile (60.9%) comprised individuals that had more proneness to present jumping to conclusions. The third profile (5.7%) presented a heterogeneous profile of metacognitive deficits. Persons with lower social cognition presented worse clinical and neuropsychological features than cluster 2 and cluster 3. Cluster 3 presented significantly worst functioning. Our results suggest that individuals with FEP present distinct profiles that concur with specific clinical, neuropsychological, and functional challenges. Each subgroup may benefit from different interventions.

## Introduction

People with first-episode psychosis (FEP) experience deficits in social cognition^1^ and metacognition^2,3^, which compromise their abilities in thinking about their own and others’ mental activities^4^.

Social cognition refers to a broad area that includes perceiving, interpreting, and processing information for adaptive social interactions^5^. There is consensus that social cognition is composed of four subdomains^6^: emotional processing refers to the ability to perceive and use emotions. Theory of mind (ToM) is the ability to attribute and represent mental states of others. Social perception encompasses decoding and interpreting social cues in others, and Attributional Bias refers to the explanations an individual gives to social events and interactions.

Metacognition refers to “thinking about thinking”^3^. One of the many domains that fall under the umbrella of metacognition is cognitive insight, which refers to the set of cognitive processes that permit questioning one’s beliefs and appraisals, and re-evaluating anomalous experiences and misinterpretations^7^. Other metacognitive constructs include cognitive biases, such as the Jumping to Conclusions (JTC) bias, which refers to the tendency of hasty decision-making. Given their role in the etiology and maintenance of psychosis, these have been thoroughly studied^3^.

Deficits in social cognition and metacognition are not a consequence of neurocognitive impairment^8,9^, but seem to be characteristics of the disorder^5,10,11^. Interestingly, social cognition and metacognition are being increasingly studied due to their contribution to functional outcome^12–16^ and negative symptoms^17,18^ in schizophrenia.

However, social cognition and metacognition do not only influence functional outcome. Instead, specific subdomains of each construct are uniquely associated to certain aspects of the illness, and of each other: inability to take the perspective of others could impact clinical insight^19,20^, which, in turn, has been associated with depression^21^, a higher number of relapses^22^, worse social functioning^23^, and poor adherence to treatment^24^. Furthermore, understanding sarcasm is a component of ToM that has been found to be specifically impaired in those with more severe social cognitive impairment and worse functional outcome^25^.

In addition, the JTC bias is related to severe and more pervasive delusions^26^, worse neuropsychological functioning^27–29^, and more compulsory admissions^30^, whereas self-reflectivity has been uniquely associated to negative symptoms and depression^31,32^. Similarly, personalizing bias seems to be associated to making more perseverative errors in cognitive flexibility tasks^33^, whereas an externalizing attributional style for negative events is associated with persecutory and grandiose beliefs^34^.

Given its established importance, recent research has focused on developing social cognitive and metacognitive remediation programs^35–37^. These interventions have emerged as promising strategies to improve outcome^37,38^, prevent chronic illness and relapse^22,39^, and increase clinical insight^40,41^. Moreover, as deficits in social cognition and metacognition are already apparent at the ultra-high risk stage^42,43^, they hold promise for early treatment in symptoms of psychosis. These interventions have yielded some clinical benefits^35,44^, although at present their potential to improve functioning is less clear. However, a recent study found that an online social cognitive intervention based on neuroplasticity can lead to functional gains in schizophrenia^45^. Although the mechanisms of change to improve functional outcome may be similar to those in cognitive remediation, of which efficacy has been well established^46^, it is yet to be determined which persons would benefit more from them.

There are two caveats in interpreting the results of the above studies: clinical trials often present averaged results, therefore blurring whether the intervention was successful for certain individuals. Likewise, it is possible that people with FEP present different profiles of social cognitive and metacognitive performance, and thus may benefit from a specific early therapeutic strategy. One way to overcome this issue is by finding subgroups of participants with specific profiles^47^. Recent studies have tackled this issue by using data-driven methods such as profile analysis. These sophisticated statistical methods allow finding profiles of cases along the dimension of interest as they occur naturally, preventing a priori assumptions^48^.

These methods have been used to profile persons with psychosis across multiple domains^5,49,50^, including social cognition and metacognition. Grouping individuals with schizophrenia on the basis of variables of social cognition has consistently yielded three profiles according to the level of impairment^25,51–53^. Conversely, studies using profile analysis in metacognitive variables have commonly found distinct profiles of persons according to symptoms^48^ and insight and depression^4^. Lysaker et al.^48^ found that independent of symptoms, poor metacognition impedes insight. As for depression and insight, Lysaker et al.^4^ found that participants with fair insight and moderate depression reported more internalized stigma, whereas those with good insight and mild depression scored higher in social cognition and metacognitive mastery.

However, these studies were conducted with samples with chronic schizophrenia and studies examining social cognition and metacognition profiles in FEP are lacking.

Identifying whether profiles of social cognition and metacognition are apparent in persons at the early stages of psychosis may provide insights into how to direct early treatment to promote recovery and prevent functional decline. Furthermore, understanding whether different profiles of social cognition and metacognition present differences in clinical and neurocognitive variables may help in identifying what persons are at a bigger risk of chronic illness.

The current study aimed to obtain profiles of individuals with FEP on the basis of social cognition and metacognitive variables using a data-driven approach in a representative sample of participants.

With this aim, we attempted to understand whether all persons with FEP present homogeneous impairments in all the domains of both constructs.

In addition, to explore the clinical presentation of each profile, we examined differences in demographics, clinical features, and neuropsychological variables among the groups. We hypothesize that patients with FEP present different profiles of social cognition and metacognition, and that profiles will differ in clinical, functional, and cognitive variables.

## Results

### Profile solution

Using Latent Profile Analysis (LPA), we identified three variable volume, variable shape, equal orientation, and ellipsodial distribution (VEE) distinct profiles of individuals with FEP according to Bayesian information criterion (BIC = −3600.651). Of all the metacognitive and social cognitive variables studied, the classification tree identified the 85–15 condition of the Beads Task and the Hinting Task as the most relevant variables in determining the profile structure.

**Fig. 1:**
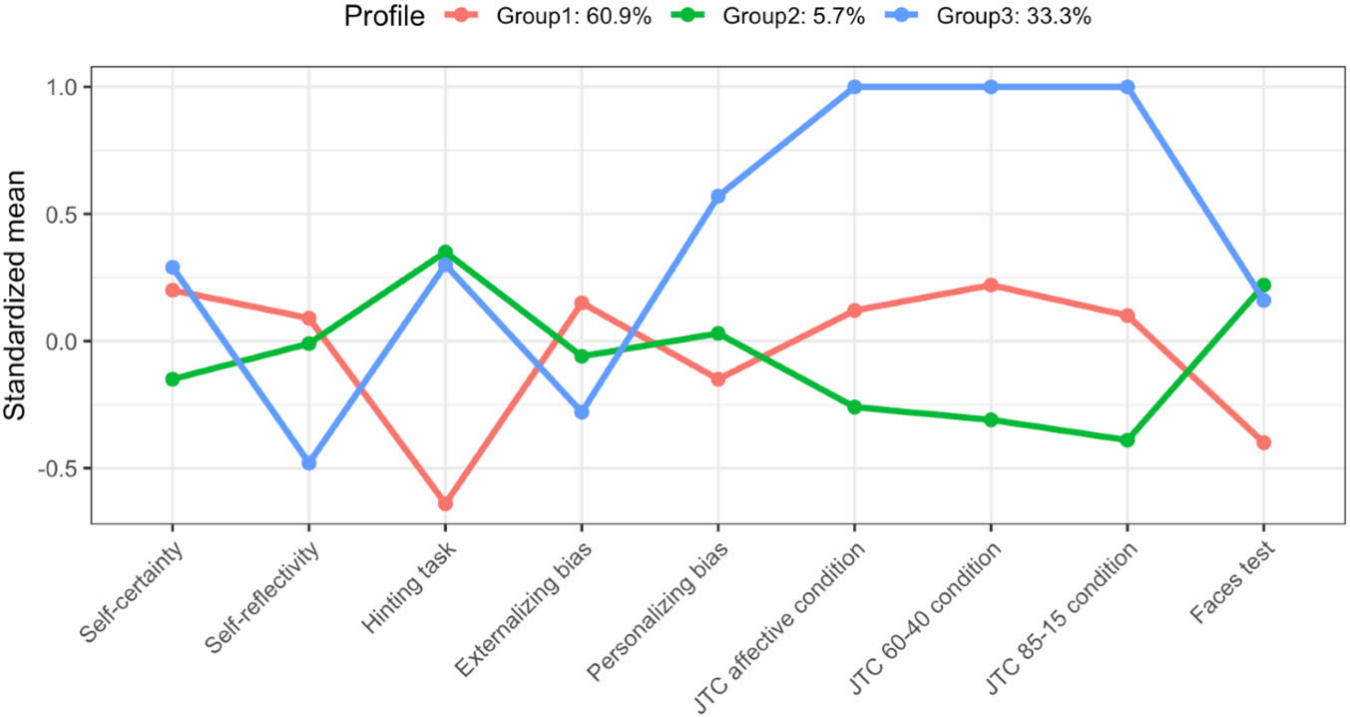
Scores of each profile in all the social cognitive and metacognitive variables included in the latent profile analysis. Values over 0 in self-certainty, self-reflectivity, externalizing bias, and personalizing bias reflect a bigger presence of the constructs. Values over 0 in the Hinting Task and the Faces Test indicate better performance in these measures. Values below 0 in the three conditions of the JTC denote more proneness to hasty decision-making.

Figure 1 describes each profile according to social cognition and metacognition variables. Table 1 summarizes the scores of the whole sample and of each profile in the social cognitive and metacognitive variables.

**Table 1:** Mean scores in the social cognitive and metacognitive variables of the whole sample and of each cluster.

	Whole sample (*N* = 174)		Cluster 1: Low S-C (*N* = 58)		Cluster 2: JTC (*N* = 106)		Cluster 3: Rigidity (*N* = 10)	
	Mean	SD	Mean	SD	Mean	SD	Mean	SD
BCIS								
Self-reflectivity^a^	15.5	4.87	16.2	5.68	15.4	4.42	13.2	3.74
Self-certainty^b^	8.33	3.39	9.00	4.06	7.87	2.86	9.30	3.83
Cognitive insight^a^	7.70	6.48	7.47	6.71	8.19	6.25	3.90	6.92
Hinting task^a^	1.58	0.38	1.30	0.48	1.73	0.23	1.70	0.18
JTC^b^								
85–15	4.88	4.30	5.52	2.75	3.14	1.56	19.6	0.69
40–60	7.90	4.96	9.14	5.43	6.34	3.43	17.3	3.65
Affective	7.57	4.55	8.22	4.77	6.40	3.38	16.3	4.16
IPSAQ^b^								
Externalizing bias	0.983	3.87	1.67	4.84	0.70	3.13	0.10	4.43
Personalizing bias	1.21	0.669	1.13	0.82	1.23	0.56	1.59	0.57
Faces test^a^	17.5	1.97	16.8	2.46	17.9	1.54	17.8	1.81

^a^Higher scores represent better ability in the construct.

^b^Higher scores represent more severity of the construct.

Profile 1 (33.3%) was characterized by prominent impairment in social cognition measures (facial emotion recognition and ToM). This profile was named “Low-SC”.

Profile 2 (60.9%) grouped participants with more proneness to JTC. We denominated this profile “JTC”. Profile 3 (5.7%) presented an excessive number of draws to decision (DTD) in the JTC tasks, higher scores in personalizing bias, more self-certainty, low self-reflectivity, and low cognitive insight. This profile was named “Rigidity”.

### Demographic, functional, and clinical characteristics

Table 2 details the demographic, functional, and clinical characteristics of the sample and of each profile. When comparing profiles, we did not find differences in age (*p* = 0.819), gender (*p* = 0.501), or years of education (*p* = 0.639). We found a trend to significance in the number of hospital admissions (*p* = 0.055), which was confirmed as significant in subsequent pairwise comparisons (Profile 1 > Profile 2).

**Table 2:** Sociodemographic, clinical, and functional characteristics of the sample and of each cluster.

	Whole sample (*N* = 174)		Cluster 1: Low S-C (*N* = 58)		Cluster 2: JTC (*N* = 106)		Cluster 3: Rigidity (*N* = 10)		Kruskal–Wallis			Cohen’s d
	Mean	SD	Mean	SD	Mean	SD	Mean	SD	*χ*^2^	*p*	DSCF^a^	
*Socio-demographics and clinical characteristics*												
Age (years)	28.1	(7.50)	27.7	(7.85)	28.2	(7.29)	28.8	(8.31)	0.400	0.819		
Gender (% female)	33.3%		31%		33%		50%		1.381	0.501		
Education (years)	13.16	4.35	12.68	4.37	13.39	4.29	13.40	5.05	0.897	0.639		
Number of admissions	1.24	1.45	1.58	1.73	1.07	1.10	1.10	0.87	5809	0.055	1–2	
Olanzapine DDD (mg)	16.94	47.26	11.46	6.23	20.73	60.31	8.88	5.27	3.608	0.165		
Comorbidities (% presence)	18.4		19%		16%		40%		3.36	0.187		
Diagnosis (%)									5.309	0.07		
Schizophrenia	39.7%		41.4%		39.6%		30%					
Psychosis (NOS)	27.6%		22.4%		32.1%		10%					
Schizoaffective disorder	10.3%		10.3%		8.5%		30%					
Delusional disorder	6.3%		8.6%		4.7%		10%					
Brief psychotic disorder	5.2%		13.8%		7.5%		10%					
Schizophreniform disorder	1.1%		3.4%		5.7%		10%					
*Clinical and functional variables*												
Emsley factors^b^												
Negative	15.4	6.95	16.8	7.30	14.5	6.70	17.5	6.36	5.741	0.057	1–2	0.323
Positive	16.1	6.40	18.7	6.91	14.7	5.77	15.2	5.47	13.591	0.001	1–2	0.599
Disorganized	8.34	3.70	9.47	4.36	7.73	3.23	8.22	2.82	7.107	0.029	1–2	0.415
Excitement	5.49	2.73	5.93	3.15	5.33	2.57	4.60	0.843	0.812	0.666		
Motor	2.86	1.45	2.91	1.61	2.82	1.34	2.90	1.66	0.136	0.934		
Depression	4.64	2.31	4.98	2.29	4.30	2.18	6.30	2.87	7.559	0.023	1–2, 1–3	0.306, 0.374
Anxiety	5.82	2.34	6.57	2.67	5.43	2.08	5.50	1.96	7.373	0.025	1–2	0.424
PANSS total score	58.42	18.39	64.88	20.96	54.79	16.29	60.10	14.59	10.43	0.005	1–2	0.50
GAF^c^	59.5	12.4	57.5	12.1	61.5	12.1	50.6	12.0	9.182	0.010	1–2, 1–3, 2–3	0.319, 0.426, 0.472
SUMD (global)^b^	6.13	3.59	7.22	3.87	2.88	3.00	5.00	3.68	7.902	0.019	1–2, 1–3	0.398, 0.43

^a^Dwass–Steel–Critchlow–Fligner pairwise comparisons.

^b^Higher scores represent more severity of the construct.

^c^Higher scores represent better ability in the construct.

We found significant differences in negative (*p* = 0.05), positive (*p* = 0.001), disorganized (*p* = 0.02), depressive (*p* = 0.02), and anxiety (*p* = 0.02) symptoms. Pairwise comparisons indicated that the Low-SC profile achieved higher scores in all the variables, indicating worse symptoms. Similarly, there were significant differences among the groups in the Scale Unawareness of Mental Disorders (SUMD). The “Low-SC” group had significantly less clinical insight.

Finally, we found significant differences among the profiles in the Global Assessment of Functioning (GAF) (*p* = 0.010). Participants in the “Rigidity” profile had significantly worse functioning than their counterparts.

### Neuropsychological characteristics of each profile

Supplementary Table 1 details the neuropsychological characteristics of the sample. The “Low-SC” group was significantly more impaired in working memory (*p* = 0.039) and in immediate recall (*p* = 0.037) than the other two profiles. We did not find any other differences among the profiles in any other neuropsychological variable.

## Discussion

In this work, we derived three distinct profiles of individuals with FEP based on social cognition and metacognition measures. LPA analysis is a statistical method that does not model potential noninvariance across latent profiles. The sensitivity of this method permitted detecting three cohesive and clinically meaningful groups of persons with FEP. Each group presented specific clinical, neuropsychological, and functional correlates.

We found a group with more prominent deficits in social cognition measures (“Low-SC”), namely Facial Emotion Recognition and ToM, another group that had a bigger tendency to present the JTC bias, and a group with worse cognitive insight scores and higher personalizing bias (“Rigidity”). The “JTC” profile had better clinical state and better neuropsychological functioning than the other two groups. The “Low-SC” profile had significantly more symptoms and worse neuropsychological functioning, whereas the “Rigidity” profile had the worst measures in functioning in the absence of demographic or clinical differences. Members of this profile exhibited lower scores in cognitive flexibility.

To the best of our knowledge, this is the first work exploring profiles of individuals on the basis of social cognition and metacognition in people with FEP. Previous studies on social cognition measures had consistently found that persons with schizophrenia can be profiled according to their level of impairment^25,51–53^. Those with worse social cognition were older, had less academic background, and were more neurocognitively impaired^51,52^. Our results are consistent with these studies in that the “Low-SC” group had worse neuropsychological performance. We did not find differences in age and education, possibly because previous studies included participants with chronic schizophrenia.

Literature examining profiles on the basis of metacognition used measures of depression and insight^4,48^, therefore non-comparable to ours. However, in a similar approach to ours, Lysaker et al.^21^ used principal component analysis to determine whether social cognition and metacognition are independent, finding clear evidence for two different factors that had specific correlations with different outcomes. The results of our study support the notion that social cognition and metacognition are two independent constructs, as we obtained two profiles based either in metacognitive variables or in variables of social cognition. It is worth noting that the “Rigidity” profile encompasses metacognitive variables and attributional style, giving support to Buck et al.^54^ who found that attributional style loaded in a distinct factor from the rest of social cognitive variables. However, understanding how social cognition and metacognition interact and what type of patient may be more prone to developing more conspicuous deficits in one of the domains in the early phases of the disorder remains to be studied.

Lysaker et al.^4^ found that participants with worse social cognition had more negative symptoms, poorer education, and poorer premorbid functioning. Conversely, individuals with poor metacognitive awareness were associated with disorganized symptoms, frequency of social contacts, and flexibility in abstract thought. Consistent with their results, we found that our profiles did not differ in age or education. We note that our sample, which demonstrated more severe social cognition impairments, had more positive, negative, and disorganized symptoms. It is likely that differences between the two studies are due to differences in measurement and in the sample, as we used different tasks and their study was conducted in a sample with established schizophrenia.

As social cognition seems to be a stable trait of the disorder^5^, a history of social cognitive deficits and negative social experiences may have a more pervasive impact on the subjects after onset. Interestingly, we found that the “Low-SC” profile had significantly less clinical insight than the other two but did not display significant deficits in cognitive insight. Although this effect could be a consequence of more positive and disorganized symptoms, there is compelling evidence reporting significant correlations between ToM and clinical but not cognitive insight^19,20^, which agrees with our results. A reason for this could be that deficits in social cognition may render subjects less able of taking into account others’ perspectives on illness, support, and treatment^4^. The literature suggests that to develop insight, others’ perspective when reflecting upon oneself must be taken into account^19^, because assessing abnormalities of one’s beliefs and perceptions require adopting not only first-person perspective but also third person, including mental health professionals’ views on treatment advice^9^.

Poor metacognition has been linked to poor outcome^55^. Specifically, the JTC bias has been associated to an increased presence of delusions^26^, worse neuropsychological functioning, and lower IQ^27–29^. We did not find these results in our “JTC” profile, although it is likely that using the number of DTD instead of a categorical variable (presence/absence of JTC) can account for the differences in our results. An alternative explanation could be that more preserved social cognition may have allowed this subset of the sample to have better premorbid adjustment, ultimately buffering the impact of the disease and fostering recovery.

The “Rigidity” group presented a heterogeneous profile that comprised specific metacognitive impairments. One of the most conspicuous traits of this profile is the excessive number of DTD in all the conditions of the Beads Task. Moreover, this group exhibited more self-certainty, lower self-reflectivity, less externalizing bias, and more personalizing bias than their counterparts, suggesting worse overall cognitive awareness. This profile could be compatible with a rigid cognitive style, in which individuals may tend to attribute negative events to other persons. Paired with more self-certainty, this group could have difficulties in realizing their interpretations are wrong and their lack of self-reflectivity could perpetuate wrong attributions. Another interpretation could be an excessive metacognitive monitoring, in the sense that subjects may be constantly evaluating whether they have enough information to make a decision. Excessive metacognition could inhibit decision-making, such as in obsessive-compulsive disorder^56^. This hypothesis could explain the remarkably high DTD in this group.

However, this group obtained significantly lower scores in Weschler Adults Intelligence Scale (WAIS-III) Digits and clinically lower scores in attention. Previous results reported that self-reflectivity is not significantly associated to most neuropsychological domains^9^, suggesting that poor self-reflectivity and neurocognitive domains may act through different pathways.

It would be plausible that participants with worse attention and less cognitive flexibility may need more information to effectively solve a problem, whereas poor self-reflectivity may compromise the subject’s ability to synthesize and comprehend ideas. The interaction of both could diminish the patient’s ability to incorporate new ideas into their self. This explanation is in agreement with findings by Berry et al.^33^ who reported an association between personalizing bias and perseverative errors.

Recent evidence has highlighted that self-certainty influences dichotomous thinking in interpersonal thinking, whereas poor self-reflectivity could diminish the differentiation between the self and others^32^. In turn, poor synthetic metacognition could increase negative symptoms^57^. As self-reflectivity allows persons to choose how to adapt to significant changes in life, such as a mental illness^3^, high self-reflectivity may protect subjects from the impact of depressive symptoms^58^, which suggests a possible link between low self-reflectivity and high depression in this profile.

There are clinical implications to our work. Persons with psychosis already present specific profiles of social cognition and metacognition at the first stages of the illness. Therefore, early treatment to the individuals’ specific needs could be delivered soon after the first episode, when persons are more amenable to treatment. Although we found neurocognitive differences among the profiles, these differences are somewhat limited and do not suggest that cognitive remediation should be tailored to specific profiles of social cognition and metacognition. Instead, the “Low-SC” profile may benefit both from specific social cognition interventions together with cognitive remediation programs. However, participants in the “Rigidity” and “JTC” profiles could be more responsive to metacognitive training programs such as the metacognitive treatment (MCT)^36,37^. Profile 3 (“Rigidity”) only grouped 5.7% of the sample. Although a small proportion of the sample, individuals in this group presented specific social cognitive and metacognitive characteristics that grant further research, as these individuals may be subject to more functional decline. Future studies should conduct clinical trials assessing the efficacy of each program in each patient profile.

Premorbid adjustment and course of the disorder may differ between the groups, and it remains to be determined what variables predict profile membership, as well as exploring differences in their course of illness. Likewise, strategies to place an individual in their corresponding profile according to their performance in measures of social cognition and metacognition are encouraged.

There are limitations in light of which our work must be interpreted. The cross-sectional design of our study precludes us from testing causality. An important limitation to our study is that the only measure of functioning is the GAF. Although widely used in research, it fails to cover all nuances of functional outcome, as it is a general measure. We did not use a healthy control group. Therefore, the extent of the impairment in each variable is unknown. We did not re-test our sample to test the stability of each profile, nor did we test our profile solution in an independent sample. The third profile comprised only ten subjects. Although we used non-parametric tests, it is possible that the statistical power was not enough to detect all the relevant differences. Finally, this work selected some commonly accepted and validated measures of metacognition and social cognition. However, metacognition encompasses a broader number of subdomains (for instance, decentration and mastery^59^), which have proven to be important therapeutic targets^60^. Future research should explore profiles of patients including more measures of metacognition.

Overall, our results indicate that individuals with FEP do not present homogeneous deficits in social cognition and metacognition, but present different profiles of performance that have an impact in their clinical presentation. Understanding the clinical course of each profile and whether they respond differentially to targeted therapies could pose clinical advances in the early treatment of psychosis.

## Methods

The design of the study is based on two research sources aimed to address the effectiveness of MCT in people with FEP, under the register numbers NCT04429412 and NCT02340559. The protocol for both studies can be accessed at https://clinicaltrials.gov/. For the purpose of this study, we only used the baseline measures of each clinical trial.

### Participants

The participants were 174 individuals with FEP. Participants were referred by their psychologists and psychiatrists at one of the community mental health services provided by the participant groups: Fundación Jiménez Díaz (Madrid), Servicio Andaluz de Jaén, Servicio Andaluz de Málaga, Centro de Salud Mental de Corporació Sanitària i Universitària Parc Taulí (Sabadell), Hospital del Mar, Consultas externas del Hospital de Sant Pau (Barcelona), Centro de Higiene Mental Les Corts (Barcelona), Hospital Universitari Institut Pere Mata (Reus), Institut d´Assistència Sanitària Girona, Hospital Clínico de Valencia, and Parc Sanitari Sant Joan de Déu (PSSJD).

Inclusion criteria were as follows: (1) a diagnosis of schizophrenia, psychotic disorder not otherwise specified, delusional disorder, schizoaffective disorder, brief psychotic disorder, or schizophreniform disorder (according to DSM-IV-TR); (2) <5 years from the onset of symptoms; (3) a score ≥ 3 in item delusions, grandiosity, or suspiciousness of Positive and Negative Syndrome Scale (PANSS) in the last year; (4) clinical stability in the previous 3 months, and (5) age between 18 and 45 years.

Exclusion criteria included (1) traumatic brain injury, dementia, or intellectual disability (premorbid IQ ≤ 70); and (2) substance dependence.

Each participant was assessed at the site by an experienced member of the study. All examiners had been previously trained to reach satisfactory concordance indexes.

### Instruments

#### Sociodemographic questionnaire

Data on sociodemographic variables, medical records, and medication were collected at the site with a questionnaire created ad hoc. We transformed the antipsychotic treatment to olanzapine defined daily dose^61^.

#### Clinical measures

The PANSS^62,63^ was used to measure clinical and general symptoms. We used the seven-factor solution proposed by Emsley et al.^64^. This solution was proven to be as sound as the five-factor model, but separates anxiety and depression into two different factors, and includes a motor factor. The Spanish version of the SUMD^65,66^ was used to measure unawareness of the mental disorder. *Metacognition*: The Beck Cognitive Insight Scale (BCIS)^67,68^ was used to measure cognitive insight. The BCIS includes two subscales that measure self-reflectivity and self-certainty, and a composite index (cognitive insight). The Beads Task^69^ was used to measure the JTC. Participants are shown two jars containing beads in two colors and in opposite ratios (85 : 15 and 60 : 40). The computer randomly selects one of the jars. Participants can either guess the jar the beads are coming from or request more beads. There is a third condition (60 : 40 ratio) in which participants extract positive and negative adjectives instead of colored beads (affective). Our outcome variable was the number of DTD. Fewer draws to decisions reflect higher proneness to jump to conclusions.

#### Social cognition

The Internal, Personal, and Situational Attributions Questionnaire (IPSAQ)^70^ was used to assess attributional style. The IPSAQ yields two subscales: externalizing bias and personalizing bias. The Faces Test^71,72^ was used to measure facial emotion recognition. A reduced version of The Hinting Task^73^ was used to measure ToM. Our reduced scale is based on the items that reached better internal consistency in the Spanish validation^74^, as the reliability of the whole scale did not reach satisfactory values. We used two research sources in this work: a subset of the sample was assessed with three stories at test and different stories at re-test to prevent learning effects. The other subset was assessed with six stories. To calculate a composite measure of the Hinting Task, we divided the total in each condition by the number of items of the test, yielding a measure between 0 and 2.

#### Functional outcome

The GAF^75^ was used to measure clinical and social functioning on a scale of 0–100.

#### Neuropsychology

The Wisconsin Sorting Card Test^76,77^ was used to assess cognitive flexibility, inhibition, strategic planning, and perseverative behavior. For the purpose of this study, we included measures of errors, perseverative errors, and non-perseverative errors. The Stroop Test^78^ was used to measure selective attention, processing speed, and resistance to interference. In this work, we have included the measure of interference converted into T-scores. The Trail Making Test (TMT-A and TMT-B)^79,80^ was used as a measure of visuomotor attention, sustained attention, speed, and cognitive flexibility. The TMT T-scores were obtained by subtracting the mean of the whole cohort to the direct punctuation, dividing it by the SD of the whole cohort, multiplying the result by 10 and adding 50. We used two research sources in this work. Part of our sample was assessed with the Continuous Performance Test (CPT-II for Windows)^81^. The other subset was assessed with the MATRICS CPT^82–84^. To obtain a homogeneous measure of attention, we created the composite variable “Attention” by adding the D-prime scores of both measures standardized into T-scores with a mean of 50 and a SD of 10. The WAIS-III^85^ subtests Vocabulary and Digits were used to measure premorbid intelligence, and verbal fluency and working memory, respectively. We obtained premorbid IQ by multiplying the scaled scores in the Vocabulary subtest by 5 and adding 50. We assessed verbal memory with the Complutense Verbal Learning Test (TAVEC)^86^. This study included the subdomains of immediate recall, effect of primacy, long-term recall, recognition, and discrimination.

### Ethics

Participants were given an informative sheet and all of them signed an informed consent file for participation in this study. The protocol of this project was approved by The Ethics Committee of Sant Joan de Déu Research Institute (Comité de Ética de Investigación con medicamentos (CEIm). The authors assert that all procedures contributing to this work comply with the ethical standards of the relevant national and institutional committees on human experimentation and with the Helsinki Declaration of 1975, as revised in 2008.

### Statistical analysis

We used SPSS Version 22 to conduct descriptive and comparative analyses. LPA was carried out using R Version 3.5.3 (R package *mclust)*. This method identifies profiles of individuals, called latent profiles, based on responses to a series of continuous variables. We determined the number of latent profiles analyzing two to six group models. The variables included were: Faces Test (total score), the Hinting Task (total score), the IPSAQ (personalizing bias and externalizing bias scores), the BCIS (self-reflectivity and self-certainty scores), and the three conditions of the Beads Task (DTD). The mean score of each variable was standardized prior to the analysis.

We determined the optimal number of latent trajectories according to the BIC^87^. We assessed the variable importance using a classification tree via the R package *rpart*. We used Kruskal–Wallis to assess mean differences in demographic, clinical, and neuropsychological variables among the profiles. We used Dwass–Steel–Critchlow–Fligner pairwise comparisons to explore the direction of the differences among groups. We calculated *U* Mann–Whitney tests between the significant pairs to obtain the effect size, transforming the statistics to obtain Cohen’s *d*.

### Reporting summary

Further information on research design is available in the Nature Research Reporting Summary linked to this article.

## Supplementary information

Supplementary Information

Reporting Summary

## Data Availability

The data supporting this research is available upon reasonable request.

## Code availability

Custom R code is available from the first author upon request.
