# Author Correction: Persons with first episode psychosis have distinct profiles of social cognition and metacognition

**DOI:** 10.1038/s41537-021-00199-4

**Published:** 2022-02-24

**Authors:** M. Ferrer-Quintero, D. Fernández, R. López-Carrilero, I. Birulés, A. Barajas, E. Lorente-Rovira, L. Díaz-Cutraro, M. Verdaguer, H. García-Mieres, J. Sevilla-Llewellyn-Jones, A. Gutiérrez-Zotes, E. Grasa, E. Pousa, E. Huerta-Ramos, T. Pélaez, M. L. Barrigón, F. González-Higueras, I. Ruiz-Delgado, J. Cid, S. Moritz, S. Ochoa, Spanish Metacognition Group

**Affiliations:** 1 Parc Sanitari Sant Joan de Déu, Sant Boi de Llobregat (Barcelona), Barcelona, Spain; 2 Departament de Psicologia Social i Psicologia Quantitativa, Universitat de Barcelona, Barcelona, Spain; 3 Investigación Biomédica en Red de Salud Mental (CIBERSAM), Barcelona, Spain; 4 Serra Húnter fellow. Department of Statistics and Operations Research (DEIO), Universitat Politècnica de Catalunya · BarcelonaTech (UPC), Barcelona, Spain; 5 Institute of Mathematics of UPC - BarcelonaTech (IMTech), Barcelona, Spain; 6 Fundació Sant Joan de Déu, Esplugues de Llobregat (Barcelona), Barcelona, Spain; 7 Departament de Psicologia Clínica i de la Salut, Facultat de Psicologia, Universitat Autònoma de Barcelona, Bellaterra, Cerdanyola del Vallès, Barcelona, Spain; 8 Department of Research, Centre d’Higiene Mental Les Corts, Barcelona, Spain; 9 Psychiatry Service, Hospital Clínico Universitario de Valencia, Barcelona, Spain; 10 Institute of Psychiatry and Mental Health, Health Research Institute (IdISSC), Clinico San Carlos Hospital, Madrid, Spain; 11 Hospital Universitari Institut Pere Mata, Institut d’Investigació Sanitària Pere Virgili (IISPV), Universitat Rovira i Virgili, Reus, Spain; 12 Department of Psychiatry, Hospital de la Santa Creu i Sant Pau, Institut d’Investigació Biomèdica-Sant Pau (IIB-Sant Pau), Universitat Autònoma de Barcelona, Barcelona, Spain; 13 Salut Mental Parc Taulí. Sabadell (Barcelona), Hospital Universitari – UAB Universitat Autònoma de Barcelona, Barcelona, Spain; 14 Neuropsiquiatria i Addicions, Hospital del Mar, IMIM (Hospital del Mar Medical Research Institute), Barcelona, Spain; 15 Department of Psychiatry, IIS-Fundación Jiménez Díaz Hospital (Madrid), Madrid, Spain; 16 Psychiatry Service, Area de Gestión Sanitaria Sur Granada, Motril (Granada), Spain; 17 Comunidad Terapéutica Jaén Servicio Andaluz de Salud, Jaén, Spain; 18 Unidad de Salud Mental Comunitaria Malaga Norte, Málaga, Spain; 19 Mental Health & Addiction Research Group, IdiBGi, Institut d’Assistencia Sanitària, Girona, Spain; 20 Department of Psychiatry and Psychotherapy, University Medical Center Hamburg, Hamburg, Germany

**Keywords:** Health sciences, Psychosis, Schizophrenia

Correction to: *npj Schizophrenia* 10.1038/s41537-021-00187-8, published online 09 December 2021

“In this article the affiliation 4 amended ‘Serra Húnter fellow. Department of Statistics and Operations Research (DEIO), Universitat Politècnica de Catalunya BarcelonaTech (UPC), Barcelona, Spain’ and new affiliation 5 added ‘Institute of Mathematics of UPC - BarcelonaTech (IMTech), Barcelona, Spain’ for Daniel Fernandez was missing. The original article has been corrected”.

