# Supplementary material for: Persons with first episode psychosis have distinct profiles of social cognition and metacognition
Source: NPJ Schizophr. 2021 Dec 9;7:61. doi: 10.1038/s41537-021-00187-8 (PMC8660816; doi:10.1038/s41537-021-00187-8)
Supplement: Supplementary file 1 — Supplementary Information [file 41537_2021_187_MOESM1_ESM.pdf]

	Whole sample (N=174)		Low S-C (N=58)		JTC (N=106)		Rigidity (N=10)		Kruskal-Wallis			
	Mean	SD	Mean	SD	Mean	SD	Mean	SD	χ^{2*}	p	DSCF‡	Cohen's d
**TMT**												
TMT-A	65.79	19.79	68.40	23.45	64.64	18.08	62.58	12.02	0.600	0.741		
TMT-B	76.58	42.36	85.86	63.54	73.07	26.76	60.99	11.22	4.056	0.132		
**WAIS-III**												
Digits†	43.82	9.38	41.31	9.47	44.84	9.09	47.74	9.61	6.513	0.039	1-2	0.364
Vocabulary(IQ)	92.8	20.4	89.6	22.8	94.1	19	98	19.7	1.32	0.516		
**WSCT**†												
Errors	45.08	13.15	43.89	12.51	45.12	12.10	51.30	23.22	2.445	0.294		
Perseverative errors	46.18	13.15	44.60	13.00	46.44	11.85	52.50	22.70	3.064	0.216		
Non-perseverative errors	44.65	13.04	43.58	12.43	44.73	12.03	49.90	23.00	2.042	0.360		
**STROOP-**Interference†	54,14	11.15	52.77	9.57	55.35	12.03	49.00	7.79	3.767	0.152		
**Attention**†	47,41	12.56	49.05	13.00	47.15	11.71	40.45	16.45	2.880	0.237		
**TAVEC**†												
Immediate recall	41.01	10.77	38	9.21	42.7	11.28	40.3	11.01	6.61	0.037	1-2	0.409
Effect of Primacy	51.99	10.62	54.0	13.7	50.8	8.69	52.62	7.23	4.45	0.108		
Long term recall	35.29	15.59	32.5	15.30	36.84	15,50	35.51	17.31	3.71	0.156		
Recognition	39.75	19.74	41.77	17.69	41.77	17.69	41.48	15.34	3.21	0.201		
Discrimination	27.02	48.43	29.54	48.85	29.54	48.85	26.11	35.37	2.68	0.262		

Table S1. Neuropsychological characteristics of the whole sample and of each profile.

*df=1

†Presented in T scores with a mean of 50 and a standard deviation of 10.

‡Dwass-Steel-Critchlow-Fligner pairwise comparisons

Spanish Metacognition Study Group (SMSG): Acevedo A[1], Alonso-Solís A[9], Anglès J[6], Ansó L[6], Argany MA[6], Aznar A[6], Barajas A[6], Barrigón ML[12], Beltrán M[16], Birulés, I[1], Bogas JL[1], Cabezas A[8], Camprubí N[16], Carbonero M[6], Carrasco E[1], Casañas R[6], Cid J[16], Conesa E[6], Corripio I[9], Cortes P[14], de Apraiz A[1], Delgado M[13], Domínguez L[13], Escartí MJ[7], Escudero A[6], Esteban Pinos I[13], Ferrer-Quintero M[1,2,3,4], Franco C[6], Frigola-Capell E[16] , Forns L[6], García C[6], Gonzalez-Casares R[13], González-Higueras F[14], González-Montoro ML[13], González E[6], Grasa-Bello E[9], Guasp A[7], Gutiérrez-Zotes[8], Huerta-Ramos ME[1], Huertas P[13], Jiménez-Díaz A[14], Lalucat LL[6] , Legido T[11], LLacer B[7], López-Carrilero R[1], López-Frutos A[7], Lorente E[7], Luengo A[7], Mantecón N[6], Mas-Expósito L[6], Montes M[15], Montserrat C[11], Moreno-Kustner B[15], Moritz S[17], Murgui E[6], Nuñez M[1], Ochoa S[1], Palomer E[16], Peláez T[1], Planell K[16], Planellas C[16], Pleguezuelo-Garrote P[14], Pousa E[9], Renovell M[7], Rubio R[6], Ruiz-Delgado I[15], Salas-Sender M[1], San Emeterio M[6], Sánchez E[6], Sánchez-Alonso S[12], Sanjuán J[7], Sans B[6], Sió H[6], Teixidó M[6], Torres P[14], Vidiella M[6], Vila MA[16], Vila-Badia R[1], Villegas F[6].

1. Parc Sanitari Sant Joan de Déu, Sant Boi de Llobregat (Barcelona); 2. Departament de Psicologia Social i Psicologia Quantitativa. Universitat de Barcelona; 3. Investigación Biomédica en Red de Salud Mental (CIBERSAM); 4. Fundació Sant Joan de Déu, Esplugues de Llobregat (Barcelona); 5. Departament de Psicologia Clínica i de la Salut, Facultat de Psicologia, Universitat Autònoma de Barcelona, Bellaterra, Cerdanyola del Vallès, Spain. Serra Húnter fellow 6. Department of Research, Centre d'Higiene Mental Les Corts, Barcelona, Spain; 7. Psychiatry Service, Hospital Clínico Universitario de Valencia; 8. Hospital Universitari Institut Pere Mata, Institut d'Investigació Sanitària Pere Virgili (IISPV), Universitat Rovira i Virgili. Reus, Spain; 9. Department of Psychiatry, Hospital de la Santa Creu i Sant Pau, Institut d'Investigació Biomèdica-Sant Pau (IIB-Sant Pau), Universitat Autònoma de Barcelona; 10. Salut Mental Parc Taulí. Sabadell (Barcelona). Hospital Universitari – UAB Universitat Autònoma de Barcelona. 11. Neuropsiquiatria i Addicions, Hospital del Mar. IMIM (Hospital del Mar Medical Research Institute). Barcelona; 12. Department of Psychiatry, IIS-Fundación Jiménez Díaz Hospital (Madrid); 13. Psychiatry Service, Area de Gestión Sanitaria Sur Granada, Motril (Granada) 14. Comunidad Terapéutica Jaén Servicio Andaluz de Salud; 15. Unidad de Salud Mental Comunitaria Malaga Norte.; 16. Mental Health & Addiction Research Group. IdiBGi. Institut d'Assistencia Sanitària, Girona; 17. Department of Psychiatry and Psychotherapy, University Medical Center Hamburg,Hamburg (Germany)
