# Supplementary material for: Persons with first episode psychosis have distinct profiles of social cognition and metacognition
Source: NPJ Schizophr. 2021 Dec 9;7:61. doi: 10.1038/s41537-021-00187-8 (PMC8660816; doi:10.1038/s41537-021-00187-8)
Supplement: Supplementary file 2 — Reporting Summary [file 41537_2021_187_MOESM2_ESM.pdf]

nature portfolio

Corresponding author(s): Dr. Susana Ochoa, PhD

Last updated by author(s): Aug 20, 2021

Reporting Summary

Nature Portfolio wishes to improve the reproducibility of the work that we publish. This form provides structure for consistency and transparency in reporting. For further information on Nature Portfolio policies, see our Editorial Policies and the Editorial Policy Checklist.

Statistics

For all statistical analyses, confirm that the following items are present in the figure legend, table legend, main text, or Methods section.

n/a	Confirmed	
☐	☒	The exact sample size (*n*) for each experimental group/condition, given as a discrete number and unit of measurement
☐	☒	A statement on whether measurements were taken from distinct samples or whether the same sample was measured repeatedly
☐	☒	The statistical test(s) used AND whether they are one- or two-sided<br>*Only common tests should be described solely by name; describe more complex techniques in the Methods section.*
☒	☐	A description of all covariates tested
☐	☒	A description of any assumptions or corrections, such as tests of normality and adjustment for multiple comparisons
☐	☒	A full description of the statistical parameters including central tendency (e.g. means) or other basic estimates (e.g. regression coefficient) AND variation (e.g. standard deviation) or associated estimates of uncertainty (e.g. confidence intervals)
☐	☒	For null hypothesis testing, the test statistic (e.g. *F*, *t*, *r*) with confidence intervals, effect sizes, degrees of freedom and *P* value noted<br>*Give P values as exact values whenever suitable.*
☒	☐	For Bayesian analysis, information on the choice of priors and Markov chain Monte Carlo settings
☒	☐	For hierarchical and complex designs, identification of the appropriate level for tests and full reporting of outcomes
☐	☒	Estimates of effect sizes (e.g. Cohen's *d*, Pearson's *r*), indicating how they were calculated

Our web collection on statistics for biologists contains articles on many of the points above.

Software and code

Policy information about availability of computer code

Data collection	No software was used to collect data.
Data analysis	We used R version 3.5.3 and SPSS v.22

For manuscripts utilizing custom algorithms or software that are central to the research but not yet described in published literature, software must be made available to editors and reviewers. We strongly encourage code deposition in a community repository (e.g. GitHub). See the Nature Portfolio guidelines for submitting code & software for further information.

Data

Policy information about availability of data

All manuscripts must include a data availability statement. This statement should provide the following information, where applicable:

- Accession codes, unique identifiers, or web links for publicly available datasets
- A description of any restrictions on data availability
- For clinical datasets or third party data, please ensure that the statement adheres to our policy

The data supporting this research is available upon reasonable request.

Field-specific reporting

Please select the one below that is the best fit for your research. If you are not sure, read the appropriate sections before making your selection.

☐ Life sciences ☒ Behavioural & social sciences ☐ Ecological, evolutionary & environmental sciences

For a reference copy of the document with all sections, see nature.com/documents/nr-reporting-summary-flat.pdf

Behavioural & social sciences study design

All studies must disclose on these points even when the disclosure is negative.

Study description	Quantitative cross-sectional design.
Research sample	We used the baseline data of two multicentric clinical trials of which Susana Ochoa was the principal investigator and obtained funding for (NCT04429412 and NCT02340559). The original clinical trials aimed to test the efficacy of Metacognitive Training in individuals with first episode psychosis. The present article merged the baseline data of the two original databases. The first clinical trial (NCT04429412) included 122 participants with first episode psychosis. The second clinical trial (NCT02340559) included 70 participants.<br>Participants were recruited from 11 hospitals in Spain. These covered different regions in the Spanish territory. For the merged database (n=174), the mean age was 28.1(SD= 7.50). A 33% of the sample was female. These characteristics grant that the sample is representative of patients with first episode psychosis.
Sampling strategy	Randomized.
Data collection	Data collection was conducted by experienced psychologists who had previously been trained to administer the instruments for this study. We calculated Cohen's kappa prior to data collection to ensure that all the evaluators had similar criteria. All data was collected using pen and paper during an at site interview with the participants. The whole assessment took three hours. Only the researcher and the participant were present. Because the participants subsequently received metacognitive training, the researchers were blind to the experimental condition, but not to the study hypothesis.
Timing	Cohort 1: January 2012-December 2014. Cohort 2: January 2016 - December 2018.
Data exclusions	We excluded 18 participants because their data on social cognitive and metacognitive measures was incomplete. Excluding these participants was decided to ensure that the results from the latent profile analysis were accurate.
Non-participation	This work tests an overarching hypothesis from two previous databases. Because the data was already collected and patients had given consent for the use of their full data for research, no participants dropped out.
Randomization	In the original studies, patients were randomized for inclusion in the study in blocks of four from a list of random numbers in each center provided by the coordinator of the study. Because this study only used the baseline data of the original studies and it is of cross-sectional nature, we did not randomize the sample.

Reporting for specific materials, systems and methods

We require information from authors about some types of materials, experimental systems and methods used in many studies. Here, indicate whether each material, system or method listed is relevant to your study. If you are not sure if a list item applies to your research, read the appropriate section before selecting a response.

Materials & experimental systems

n/a	Involved in the study	
☒	☐	Antibodies
☒	☐	Eukaryotic cell lines
☒	☐	Palaeontology and archaeology
☒	☐	Animals and other organisms
☐	☒	Human research participants
☐	☒	Clinical data
☒	☐	Dual use research of concern

Methods

n/a	Involved in the study	
☒	☐	ChIP-seq
☒	☐	Flow cytometry
☒	☐	MRI-based neuroimaging

Human research participants

Policy information about studies involving human research participants

Population characteristics	See above

March 2021

Recruitment	Participants were recruited through their psychiatrists and psychologists.<br>Psychiatrists and psychologists that assisst persons with psychosis were explained the aims of the study and the inclusion criteria. Then, they present potential volunteers with information regarding the study, and refer them to the research unit if they accept participating in the study. Neither the PI of the study nor the research assistants that conducted the assessments work as clinicians in the same hospital, thus reducing the risk of self-selection bias.
Ethics oversight	The Ethics Committee at Institut de Recerca de Sant Joan de Déu (coordinating centre) approved this study.

Note that full information on the approval of the study protocol must also be provided in the manuscript.

Clinical data

Policy information about clinical studies

All manuscripts should comply with the ICMJE guidelines for publication of clinical research and a completed CONSORT checklist must be included with all submissions.

Clinical trial registration	NCT04429412 and NCT02340559
Study protocol	The study protocols for the original work can be found in: https://clinicaltrials.gov/ct2/show/NCT04429412?term=NCT04429412&draw=2&rank=1 and https://clinicaltrials.gov/ct2/show/NCT02340559?term=NCT02340559&draw=2&rank=1 .
Data collection	Data was collected from January 2012 to December 2018 in two cohorts (Cohort 1: January 2012-December 2014. Cohort 2: January 2016 - December 2018.)
Outcomes	For the purposes of our study, our main outcome were profiles of social cognition and metacognition. Secondary outcomes were measures of symptoms, neurocognitive performance and functioning, The outcomes of the parent studies can be consulted in the protocols.
